# Anti-inflammatory activity of aqueous extract of *Mirabilis jalapa* Linn. leaves

**DOI:** 10.4103/0974-8490.75456

**Published:** 2010

**Authors:** Manjit Singh, Vijender Kumar, Ishpinder Singh, Vinod Gauttam, Ajudhia Nath Kalia

**Affiliations:** *Department of Pharmacognosy, L.L.R. College of Pharmacy, Moga, Punjab-142001, India*; 1 *Department of Pharmacognosy, L.L.R. College of Pharmacy, Moga, Punjab-142001, India*; 2 *Department of Pharmacognosy, Adesh College of Pharmacy, Bathinda - 151001, Punjab, India*

**Keywords:** Anti-inflammatory activity, aqueous extract, *Mirabilis jalapa*, paw edema

## Abstract

**Background:**

Theobjective of the present study was to evaluate the anti-inflammatory activity of aqueous extract of *Mirabilis jalapa* Linn. (MJL)(Nyctaginaceae) leaves for scientific validation of the folklore claim of the plant. The leaves are used as traditional folk medicine in the south of Brazil to treat inflammatory and painful diseases. Cosmetic or dermo-pharmaceutical compositions containing MJL are claimed to be useful against inflammation and dry skin.

**Methods:**

Aqueous extract of the leaves was prepared by cold maceration.

**Results:**

The anti-inflammatory activity was evaluated using carrageenan and formalin-induced paw edema models in Wistar albino rats. The anti-inflammatory activity was found to be dose dependent in carrageenan-induced paw edema model. The aqueous extract has shown significant (*P* < 0.05) inhibition of paw oedema, 37.5% and 54.0% on 4 ^th^ hour at the doses of 200 and 400 mg/kg, respectively. Similar pattern of paw edema inhibition was seen in formalin-induced paw edema model. The maximum percentage inhibition in paw edema was 32.9% and 43.0% on 4 ^th^ day at the doses of 200 and 400 mg/kg, respectively.

**Conclusion:**

The results of present study demonstrate that aqueous extract of the leaves possess significant (*P* < 0.05) anti-inflammatory potential.

## INTRODUCTION

Herbal therapy, although still an unwritten science, is well established in some countries and traditions and has become a way of life in almost 80% of population in rural areas. Chronic anti-inflammatory diseases including rheumatoid arthritis are still one of the main health problems of the world’s population. At present, although synthetic drugs are dominating the market but element of toxicity that these drugs entail, cannot be ruled out. Their prolonged use may cause severe adverse effects on chronic administration[1] the most common being gastrointestinal bleeding and peptic ulcers.[2] Consequently there is a need to develop a new anti-inflammatory agent with minimum side effects. Search for safe and effective anti-inflammatory agents have been given priority in scientific research in herbal system of medicine.

*Mirabilis jalapa* Linn. (Nyctaginaceae; MJL) is known as ‘Maravilla’ or ‘Bonnia’ in Brazil, ‘Marvel of Peru’ in Peru, ‘Gulambasa’ in Ayurveda, ‘Four o’ clock’ in English, and ‘Gul-abbas’ in Hindi.[3] It is the native of tropical America but widely cultivated as a decorative plant in several other countries.[4] The leaves are used as traditional folk medicine in the south of Brazil to treat inflammatory and painful diseases and as a laxative.[3,5,6] Cosmetic or dermo-pharmaceutical compositions containing MJL are claimed to be useful against inflammation and dry skin.[7] Several components such as β-sitosterol, stigmasterol, ursolic acid, oleanolic acid, brassicasterol, and Mirabilis antiviral protein, rotenoids (mirabijalone A-D, boeravinones C and F) have been isolated from the aerial parts and roots, respectively.[5,8–10] Furthermore, different extracts are reported to have numerous biological activities viz. antispasmodic, antibacterial, antiviral, antifungal, protein synthesis inhibition, etc.[11–15] Anti-inflammatory activity of total alcoholic and petroleum ether extracts of leaves has already been proved.[16] Since water is the most common and safe solvent as compared to methanol and petroleum ether for preparing ayurvedic formulations, the present study was aimed to investigate the anti-inflammatory property of the aqueous extract of leaves.

## MATERIALS AND METHODS

### Collection of plant material

Leaves of MJL were collected in the month of June, 2008 from Tirupati and authenicated by Dr. K.M. Chetty, Assistant Professor, Department of Botany, Sri Venkateswara University, Tirupati. A voucher specimen (MLS 9) is deposited in herbarium of I.S.F. College of Pharmacy, Moga, India. The leaves were washed with water, shade dried, coarsely powdered, and kept in air tight container till use.

### Preparation of extracts and preliminary phytochemical screening

Aqueous extract was prepared by cold maceration. Extract was filtered and concentrated in rotary evaporator. The extract was dried in a vacuum desiccator to obtained constant weight. The phytochemical screening was carried out as described by Norman.[17]

### Animals

Albino Wistar rats of either sex weighing 150−200 g were obtained from Indian Institute of Integrated Medicines, Jammu. All animals were housed in polypropylene cages (3 in each cage) at an ambient temperature; 25 ± 2°C, relative humidity; 55−65%, and were maintained under a 12 h light/dark cycle each in animal house of I.S.F. College of Pharmacy, Moga. Ethical clearance for this experimental protocol was obtained from the Institutional Animal Ethics Committee (Reg.No.816/04/c/CPCSEA). The animals were fed with standard diet and water *ad libitum* and were deprived of food overnight prior to the experiment.

### Drugs and chemicals

Carrageenan was procured from Sigma Chemical Co. (St Louis, MO, USA), diclofenac injection (Voveran) from Novartis India Ltd., Bombay and formalin from Ranbaxy (Rankem). Vernier caliper purchased from Percision India Ltd. and standard chow diet from Ashirwad Industries, Ropar (Punjab) were used in the study.

### Acute toxicological evaluation

To assess the acute toxicity of MJL determination of LD _50_ value of the aqueous extract was attempted using the up-and-down method as described by Bruce.[18]

### Drug administration

The test extract was administered by suspending in 1% Carboxy methyl cellulose (CMC) solution.

In carrageenan model, aqueous extract of MJL leaves at doses of 200 and 400 mg/kg, while diclofenac sodium at dose of 10 mg/kg were administered orally using gastric canula 30 min. before the carrageenan injection in sub plantar region of rat paw.

In formalin model, the extract and standard drug were administered in the same way and at same dose as mentioned above, except that treatment continued for seven consecutive days while formalin was given only on first day.

### Evaluation of *invivo* anti-inflammatory activity and grouping of animals

#### Carrageenan-induced paw edema model

Paw edema was induced[19] by injecting 0.1 ml of 1% w/v carrageenan suspended in 1% CMC into sub-plantar tissues of the left hind paw of each rat. Rats were divided into four groups; each group consisting of six animals.

Group I Carrageenan control
Group II Aqueous extract (200 mg/kg)
Group III Aqueous extract (400 mg/kg)
Group IV Diclofenac sodium (10 mg/kg) as standard reference

The paw thickness was measured before injecting the carrageenan and after 60, 120, 180, 240 min. using vernier caliper. The anti-inflammatory activity was calculated as percentage inhibition of oedema in the animals treated with extract under test in comparison to the carrageenan control group.

The percentage (%) inhibition of edema is calculated using the formula

$$\%\;\mathrm{inhibition}\;=\;\frac{T_{o}\;-\;T_{t}}{T_{o}}\times 100$$

Where T _t_ is the thickness of paw of rats given test extract at corresponding time and T _o_ is the paw thickness of rats of control group at the same time.

### Formalin-induced paw edema model

The animals were treated in the same way as in above model except that formalin (0.2 ml of 2% v/v freshly prepared formalin solution prepared in distilled water) was used as edematogenic agent.[20]

Group I Formalin control
Group II Aqueous extract (200 mg/kg)
Group III Aqueous extract (400 mg/kg)
Group IV Diclofenac sodium (10 mg/kg) as standard reference

The thickness was measured before injecting the formalin and after injecting the formalin everyday at a fixed time for seven consecutive days using a vernier caliper (precision).

### Data Analysis

The data is expressed as mean ± Standard Deviation (SD). Results were analyzed using one-way ANOVA followed by Dunnet’s test. Differences were considered as statistically significant at P < 0.05, when compared with control.

## RESULTS

### Extraction and preliminary phytochemical screening

The yield of aqueous extract was found to be 16.2% w/w. The phytochemical screening revealed the presence of carbohydrates, proteins, amino acids, flavonoids, alkaloids, tannins, and phenolic compounds in the aqueous extract and findings were identical as reported earlier by Lakshminath *et al*.[21]

### Anti-inflammatory activity

Table 1 shows the effect of aqueous extract of leaves and standard drug as compared to carrageenan control at different hours in carrageenan-induced paw edema model using vernier caliper. Aqueous extract administered at a dose of 200 mg/kg p.o prevented carrageenan-induced paw edema with a percentage inhibition of 15.0%, 26.4%, 31.3%, and 39.0% at 1, 2, 3,and 4 hour, respectively, while 25.6%, 35.0%, 50.0%, and 56.3% at a dose of 400 mg/kg p.o. at 1, 2, 3.and 4 hour, respectively. Diclofenac sodium at a dose of 10 mg/kg p.o. prevented carrageenan-induced paw edema with a percentage inhibition of 56.0%, 66.2%, 68.9%, and 72.1% at 1, 2, 3.and 4 hour, respectively.

Table 2 shows the day wise effect of aqueous extract of leaves and standard drug as compared to formalin control group in formalin-induced paw edema model using vernier caliper. Aqueous extract administered extract prevented formalin-induced paw edema with percentage inhibition of 32.9% and 43.0% on 4 ^th^ day at doses of 200 and 400 mg/kg, respectively, while diclofenac sodium (10 mg/kg) showed 57.0% of percentage inhibition of paw edema on 4^th^ day.

**Table 1:** Effect of aqueous extract of MJL leaves at doses of 200 and 400 mg/kg, and diclofenac sodium as compared to carrageenan control group at different hours in carrageenan-induced paw edema model using vernier caliper.

Groups	Dose of extract (mg/kg) p.o.	Change in paw thickness (mm) ± SD (% inhibition)			
		1^st^ h	2^nd^ h	3^rd^ h	4^th^ h
Carrageenan control (0.1 ml of 1% w/v)	-	1.34 ± 0.1	2.37 ±0.118	3.64 ± 0.147	3.23 ± 0.161
Carrageenan(0.1 ml of 1% w/v) + aqueous extract	200	1.14 ± 0.103 (14.93%)	1.74 ± 0.217^a^ (26.44%)	2.50 ± 0.106^a^ (31.3%)	1.97 ± 0.115^a^ (39.0%)
Carrageenan (0.1 ml of 1% w/v) + aqueous extract	400	1.00 ± 0.12 (25.6)%	1.54 ± 0.163^a^ (35.02%)	1.81 ± 0.099^a^(50.09%)	1.41 ± 0.188^a^(56.34%)
Carrageenan (0.1 ml of 1% w/v) + diclofenac sodium	10	0.59 ± 0.118^a^(55.97%)	0.8 ± 0.121^a^(66.24%)	1.14 ± 0.125^a^(68.82%)	0.9 ± 0.118^a^(72.13%)

All values are expressed as mean ± SD;

a *P* < 0.05 v/s carrageenan control

**Table 2:** Day wise effect of aqueous extract of MJL leaves at doses of 200 and 400mg/kg, and diclofenac sodium as compared to formalin control group in formalin-induced paw edema model using vernier caliper.

Groups	Dose (mg/kg) p.o.	Change in paw thickness (mm) ± SD (% inhibition)						
		1^st^ day	2^nd^ day	3^rd^ day	4^th^ day	5^th^ day	6^th^ day	7^th^ day
Formalin control (0.2 ml of 2% v/v)	-	4.90±0.20	3.90±0.22	3.16±0.24	2.62±0.17	2.00±0.19	1.59±0.25	1.44±0.26
Formalin (0.2 ml of 2% v/v) + aqueous extract.	200	4.16±0.21^a^ (15.10%)	3.05±0.19^a^ (21.89%)	2.31±0.23^a^ (26.94%)	1.76±0.20^a^ (32.88%)	1.45±0.23^a^ (27.6%)	1.21±0.26^a^ (23.87%)	1.15±0.25(19.90%)
Formalin (0.2 ml of 2% v/v) + aqueous extract	400	3.82±0.22^a^ (22.12%)	2.76±0.18^a^ (29.23%)	1.99±0.23^a^ (36.83%)	1.44±0.19^a^ (43.0%)	1.23±0.24^a^ (38.3%)	1.05±0.27^a^ (33.62%)	1.02±0.24^a^ (29.17%)
Formalin (0.2 ml of 2% v/v) + diclofenac sodium	10	3.53±0.19^a^ (28.03%)	2.46±0.15^a^ (36.88%)	1.71±0.18^a^ (46.08%)	1.16±0.16^a^ (55.73%)	1.22±0.15^a^ (43.91%)	0.97±0.14^a^ (38.91%)	0.95±0.17^a^ (33.45%)

All values are expressed as mean ± SD;

*P* < 0.05 v/s formalin control

## DISCUSSION

Carrageenan-induced acute inflammation is one of the most suitable test procedure to screen anti-inflammatory agents. The time course of edema development in carrageenan-induced paw edema model in rats is generally represented by a biphasic curve.[22] The first phase of inflammation occurs within an hour of carrageenan injection and is partly due to the trauma of injection and also due to histamine and serotonin component.[23] As shown in Table 1, there was no significant inhibition of paw edema, 15.0% and 25.6% in the early hours of study by aqueous extract at 200 and 400 mg/kg, respectively. Hence, it can be concluded that there is no inhibition of histamine and serotonin. Carrageenan-induced paw edema model in rats is known to be sensitive to cyclo-oxygenase inhibitors and has been used to evaluate the effect of non-steroidal anti-inflammatory agents, which primarily inhibit the cyclo-oxygenase involved in prostaglandin synthesis.[24] It plays a major role in the development of the second phase of inflammatory reaction, which is measured at the 3^rd^ hour.[25] As shown in the Table 1, there is a significant (*P* < 0.05) percentage inhibition of paw edema, 31.3% and 50.1% at doses of 200 and 400mg/kg, respectively, at 3^rd^ hour by aqueous extract. Therefore, it can be inferred that the inhibitory effect of aqueous extract on carrageenan-induced inflammation may be due to inhibition of the enzyme cyclo-oxygenase leading to inhibition of prostaglandin synthesis.

Formalin-induced paw edema is one of the most suitable test procedure to evaluate chronic anti-inflammation, as it closely resembles human arthritis.[26] As shown in Table 2, administration of aqueous extract prevented formalin-induced paw edema in a dose-dependent manner showing significant anti-inflammatory effect on 4^th^ day, percentage inhibition shown was found to be 32.9% and 43.0% at dose of 200 and 400 mg/kg, respectively. Hence, it is suggested that aqueous extract of MJL leaves may provide benefits in the management of arthritis.

## CONCLUSION

Aqueous extract of MJL possess significant anti-inflammatory potential. These findings support the use of the extract in traditional system of medicine for the management of inflammatory conditions.
